# Sharing of either phenotypes or genetic variants can increase the accuracy of genomic prediction of feed efficiency

**DOI:** 10.1186/s12711-022-00749-z

**Published:** 2022-09-06

**Authors:** Sunduimijid Bolormaa, Iona M. MacLeod, Majid Khansefid, Leah C. Marett, William J. Wales, Filippo Miglior, Christine F. Baes, Flavio S. Schenkel, Erin E. Connor, Coralia I. V. Manzanilla-Pech, Paul Stothard, Emily Herman, Gert J. Nieuwhof, Michael E. Goddard, Jennie E. Pryce

**Affiliations:** 1 Agriculture Victoria Research, Agribio, Bundoora, VIC 3083 Australia; 2 Agriculture Victoria Research, Ellinbank Centre, Ellinbank, Gippsland, VIC 3821 Australia; 3 School of Agriculture and Food, University of Melbourne, Parkville, VIC 3010 Australia; 4 LACTANET, Sainte-Anne-de-Bellevue, QC H9X 3R4 Canada; 5 CGIL, University of Guelph, Guelph, ON N1G 2W1 Canada; 6 Institute of Genetics, Vetsuisse Faculty, University of Bern, 3002 Bern, Switzerland; 7 Animal Genomics and Improvement Laboratory, USDA, Agricultural Research Service, Beltsville Agricultural Research Center, Beltsville, MD 20705 USA; 8 Department of Animal and Food Sciences, University of Delaware, Newark, DE 19716 USA; 9 Center for Quantitative Genetics and Genomics, Aarhus University, Blichers Alle 20, 8830 Tjele, Denmark; 10 Faculty of Agricultural, Life & Environmental Sciences, University of Alberta, Edmonton, AB T6G 2R3 Canada; 11 DataGene Ltd, Agribio, Bundoora, VIC 3083 Australia; 12 School of Veterinary and Agricultural Sciences, University of Melbourne, Parkville, VIC 3052 Australia; 13 School of Applied Systems Biology, La Trobe University, Bundoora, VIC 3083 Australia

## Abstract

**Background:**

Sharing individual phenotype and genotype data between countries is complex and fraught with potential errors, while sharing summary statistics of genome-wide association studies (GWAS) is relatively straightforward, and thus would be especially useful for traits that are expensive or difficult-to-measure, such as feed efficiency. Here we examined: (1) the sharing of individual cow data from international partners; and (2) the use of sequence variants selected from GWAS of international cow data to evaluate the accuracy of genomic estimated breeding values (GEBV) for residual feed intake (RFI) in Australian cows.

**Results:**

GEBV for RFI were estimated using genomic best linear unbiased prediction (GBLUP) with 50k or high-density single nucleotide polymorphisms (SNPs), from a training population of 3797 individuals in univariate to trivariate analyses where the three traits were RFI phenotypes calculated using 584 Australian lactating cows (AUSc), 824 growing heifers (AUSh), and 2526 international lactating cows (OVE). Accuracies of GEBV in AUSc were evaluated by either cohort-by-birth-year or fourfold random cross-validations. GEBV of AUSc were also predicted using only the AUS training population with a weighted genomic relationship matrix constructed with SNPs from the 50k array and sequence variants selected from a meta-GWAS that included only international datasets. The genomic heritabilities estimated using the AUSc, OVE and AUSh datasets were moderate, ranging from 0.20 to 0.36. The genetic correlations (r_g_) of traits between heifers and cows ranged from 0.30 to 0.95 but were associated with large standard errors. The mean accuracies of GEBV in Australian cows were up to 0.32 and almost doubled when either overseas cows, or both overseas cows and AUS heifers were included in the training population. They also increased when selected sequence variants were combined with 50k SNPs, but with a smaller relative increase.

**Conclusions:**

The accuracy of RFI GEBV increased when international data were used or when selected sequence variants were combined with 50k SNP array data. This suggests that if direct sharing of data is not feasible, a meta-analysis of summary GWAS statistics could provide selected SNPs for custom panels to use in genomic selection programs. However, since this finding is based on a small cross-validation study, confirmation through a larger study is recommended.

**Supplementary Information:**

The online version contains supplementary material available at 10.1186/s12711-022-00749-z.

## Background

The dairy industry has seen tremendous gains by selecting for milk production yield, while maintenance requirements have increased more slowly, leading to improvements in gross efficiency (defined as production per unit of intake) [1]. However, feed costs still make up a large proportion of the variable and total costs on a dairy farm, and improving production efficiency is therefore a key breeding objective [2]. Feed efficiency, as a measure of converting feed into additional volumes of milk solids, is an important topic in the era where food demand is increasing with the growing human population. In order to increase the overall gross production efficiency of livestock, it is important to develop accurate genomic breeding tools that improve or maintain feed efficiency in parallel with progress in production, health, and fertility.

Over the last 20 years, the Australian dairy industry has had a penalty on maintenance in national selection indices, initially through the inclusion of body weight, and more recently through “Feed Saved”. The Feed Saved trait accounts for maintenance requirements through body weight in addition to metabolic efficiency through residual feed intake (RFI). RFI is defined as the difference in an animal’s actual and expected dry matter intake (DMI), after adjusting for its body size, growth, and productivity. Variation in RFI is likely due to differences between animals in the metabolic processes related to feed intake level, digestion of feed, absorption of nutrients, basal metabolism, health status, rumen microbial metabolism, and physical activity [3–5].

Genomic selection has been widely adopted by the dairy industry and is especially useful for traits such as RFI that are very expensive and difficult-to-measure. The accuracy of genomic estimated breeding values (GEBV) depends on the size of the training population and the extent of linkage disequilibrium between single nucleotide polymorphisms (SNPs) and causal variants [6]. Due to the high costs of measuring RFI, most countries have insufficient amounts of data to achieve high accuracy of GEBV. For example, the accuracies of genomic predictions for RFI and DMI have been reported to be around 0.2 to 0.4 in beef and dairy cattle studies [5, 7–14] using reference populations that vary in size between 527 and 7k individuals. Studies on beef cattle have the additional challenge of multi-breed reference populations: for example Bolormaa et al. [8] estimated a genomic prediction accuracy of 0.36 for RFI using 4k animals from nine breeds of *Bos taurus* and *Bos indicus* cattle.

Combining data from international partners on feed intake may be one way to increase the quantity of data available to improve the accuracy of GEBV. Furthermore, the data could be used to increase the power and precision of mapping the causal variants for feed efficiency from imputed sequence data. However, collating a large enough training population for accurate GEBV prediction is a major challenge for feed efficiency traits due to different management systems, which can lead to the occurrence of genotype-by-environment interactions between countries and continents. Furthermore, genetic differences may exist between traits and measurements that are taken at different stages of the lactation period. Nonetheless, the study by Berry et al. [15] successfully collated DMI records for about 9k dairy animals from nine countries to estimate genetic parameters. These collaborative initiatives continue, such as with the Efficient Dairy Genome Project (EDGP), that was established with the goal of allowing free access among partners to a large training population of Holstein dairy cows for feed intake, body weight, and milk production.

Genomic selection refers to selection decisions that are based on breeding values predicted using genome wide marker data such as SNPs [16]. Genomic best linear unbiased prediction (GBLUP) method, where SNP information is incorporated in the BLUP method, is currently used for routine genomic evaluation in Australia. GBLUP assumes that all SNP effects are drawn from the same normal distribution and, therefore, all SNPs have small effects. A multi-trait GBLUP model is expected to improve the accuracy of GEBV with the help of information from genetically correlated traits. Therefore, testing the accuracy of genomic prediction using Australian cow RFI data as either an individual trait, or a correlated trait with the RFI data of international cows and Australian heifers would be of interest.

Until recently, the Australian dairy industry relied mainly on the genotypes obtained with the Illumina Bovine 50k SNP panel for genomic prediction of breeding values. However, standard SNP arrays (e.g., 50k SNPs) are unlikely to include causal variants because they are composed of random markers that are preselected only to be (highly) polymorphic across breeds. This can result in less common causal variants not being in strong linkage disequilibrium (LD) with the SNPs on the array; thus, their effects may not be captured in genomic predictions. Indeed, there is now evidence in both cattle and sheep that the accuracy of genomic prediction can be increased by combining several thousands of highly predictive sequence variants using a standard SNP array [17–21]. In contrast to standard SNP arrays, whole-genome sequence (WGS) data should include many of the less common causal variants. In addition, the use of these selected sequence variants that are causal or very close to the causal variant are particularly beneficial for prediction of animals that have a low relationship to the reference animals. Thus, it is of considerable interest to undertake a meta-analysis of GWAS with imputed sequence variants (e.g., sharing the signed-t values of sequence variant effects) as an alternative to sharing the raw data and genotypes among countries. The resulting GWAS could be used to fine-map putative causal variants and genes that underlie feed intake and efficiency. Furthermore, addition of variants with large effects for single traits or with multiple pleiotropic effects to custom SNP panels is of particular interest to improve the accuracy of genomic prediction for feed efficiency.

The main objectives of our study were: (1) to improve the accuracy of genomic prediction for RFI of Australian cows by treating their RFI records as either a correlated trait with international cow and/or Australian heifers’ RFI records or a same trait as a combined dataset; and (2) to increase the accuracy of genomic prediction for feed efficiency in Australian cows by augmenting the standard 50k SNP panel with a subset of sequence variants selected from GWAS using international cow data.

## Methods

### Phenotypes

In this study, we used feed intake, body weight, and milk production data of the overseas cows from research groups in seven countries (Australia (AUS), United States of America (USA), Canada (CAN), Denmark (DNK), United Kingdom (GBR), Switzerland (CHE), and the Netherlands (NLD)) (Table 1). The combination of the USA and CAN datasets is referred to as the North American (NA) dataset, the combination of the remaining international countries as the European (EU) dataset, and the combination of the NA and EU datasets as the overseas dataset (OVE).

**Table 1:** List of datasets and GBLUP analyses used in this study

Dataset code	Analyses^a^	Heifer or cow	Country or continent
AUSh	Univariate	Growing heifer	Australia
AUSc	Univariate	Lactating cow	Australia
USA	na	Lactating cow	United States
CAN	na	Lactating cow	Canada
DNK	na	Lactating cow	Denmark
GBR	na	Lactating cow	United Kingdom
CHE	na	Lactating cow	Switzerland
NLD	na	Lactating cow	the Netherlands
EU	Univariate	Lactating cow	DNK + GBR + CHE + NLD
NA	Univariate	Lactating cow	USA + CAN
OVE	Univariate	Lactating cow	EU + NA
AUShAUSc	Bivariate	Heifer + cow	AUSh and AUSc
AUScEU	Bivariate or univariate	Lactating cow	AUSc and EU
AUScNA	Bivariate or univariate	Lactating cow	AUSc and NA
AUScOVE	Bivariate or univariate	Lactating cow	AUSc, AUSc and OVE
AUShAUScEU	Trivariate	Heifer + cow	AUSc, AUSc and EU
AUShAUScNA	Trivariate	Heifer + cow	AUSc, AUSc and NA
AUShAUScOVE	Trivariate	Heifer + cow	AUSc, AUSc and OVE

^a^na: not applicable; bivariate or trivariate: analyses using two or three datasets, which were treated as individual traits, respectively

#### Australian heifer (AUSh)

The AUSh dataset comprised a total of 824 Holstein heifers, which were measured for residual feed intake (RFI, kg/days), dry matter intake (DMI, kg/d), mean body weight (BW, kg), and change in body weight (∆BW, kg), and the phenotypes were adjusted for systematic environment effects (experiment), age at measurement, and its squared value. Trait deviations for RFI in AUS heifers were previously calculated as mean values of the difference in actual and predicted DMI that was measured over a 6 to 7-week period on about 6-month old heifers. A description of the design, measurements, and calculations for RFI is in Pryce et al. [22]. Full details of the management of animals, the diets they were fed, and recording of each trait are described in Williams et al. [23] and Pryce et al. [22].

#### Australian cow (AUSc)

The AUSc dataset consisted of 584 cows, of which 137 overlapped with the animals in the AUSh dataset (i.e., they had measurements as heifers and as cows) [22]. RFI for AUSc was calculated based on the average DMI over the 28-days experimental period using the model that is described in Pryce et al. [22], which is as follows: RFI = DMI –  (mean + contemporary group + DIM + Parity + ECM + MBW + ∆BW), where mean is the overall mean of DMI across the population, MBW is the mean BW, and ∆BW is the change in BW during the trial period, ECM is the energy-corrected milk; ECM, MBW, and ∆BW were fitted as covariates in the model; contemporary group (16 different cohort groups from trials run between November 2011 and November 2017), DIM at the beginning of the trial as a covariate, and parity (1, 2, 3, and 4+) were the systematic environmental effects fitted as fixed effects. Units for traits in the model are the same as in the AUSh data. The RFI values were averaged when more than one lactation record was available per animal. Energy-corrected milk (kg/days) was calculated as in Pryce et al. [22]: ECM = 0.1 × milk (kg/days) + 5.2 × fat (kg/days) + 2.6 × protein (kg/days), where milk, fat, and protein are milk, protein, and fat yields, respectively.

#### Overseas cows

The phenotypes from USA, CAN, DNK, and CHE were downloaded from the EDGP database in December 2019. The RFI phenotypes from NLD and GBR are described in Pryce et al. [22] and the DMI phenotypes for GBR were extracted from the EDGP database. Initially the dataset of each country was treated separately. The number of records and animals for RFI and the traits used to calculate RFI are in Table 2. The RFI phenotypes for OVE cows within each country were calculated as RFI = DMI – (mean + parityST + DIM + HYS + poly(age,-2) + trial + ECM + MBW + ∆BW), where DMI, ECM, MBW, and DIM are the same as above, ∆BW is daily BW change, and poly(age,-2) is the age of cows at calving fitted as a second-order orthogonal polynomial; ∆BW was calculated by fitting a fifth-order orthogonal polynomial regression on DIM (5 to 306 DIM) to daily BW, and then ∆BW was calculated as the difference in predicted BW between consecutive days. Units for traits in the model are the same as for the AUSh data. In the above model, trial was only fitted for the CHE data, which covered three different feeding systems. ECM for each overseas country was calculated using the same formula [22] that was used to calculate ECM for AUSc. To calculate phenotypes of RFI, no missing records were allowed for the covariates ECM, BW, and ∆BW (Table 2). The fixed effects were: overall mean across population (mean), parity stage (parityST), and herd-year-season (HYS). For CHE, diets (3 levels) were also fitted as fixed effects. ParityST is a combination of parity (3 levels: 1, 2, and 3+) and four stages of lactation (4 levels: $\le$ 30 days, 31–100, 101–200; and $>$ 200 days), and HYS is a combination of herd, year, and season of calving (2 herds for CAN, 3 herds for CHE, and the remaining countries had animals from one herd, and four seasons (autumn (October 1–December 31), winter (January 1–March 31), spring (April 1–June 30), and summer (July 1–September 30)). Means for each trait were calculated per animal. Trait deviations for RFI in NLD and GBR cows were calculated in an earlier study by Pryce et al. [22], which also provides a complete description of the design, measurements, and calculations. Before collating the datasets from each country, each dataset was standardised ((x − mean)/SD) to avoid any potential differences in measurement scales, and country of origin was always fitted as a fixed effect in further analyses.

**Table 2:** Number of records (and number of animals in parentheses) for dry matter intake and traits used to calculate residual feed intake per country

Dataset	DMI	ECM	BW	∆BW	DMI_RFI_
AUSh	46,144 (824)		46,144 (824)	46,144 (824)	46,144 (824)
AUSc	20,384 (584)	20,384 (584)	20,384 (584)	20,384 (584)	20,384 (584)
USA	126,863 (673)	18,485 (676)	127,693 (672)	127,693 (672)	17,568 (671)
CAN	137,517 (755)	38,543 (785)	150,208 (492)	150,208 (492)	13,278 (473)
DNK	34,284 (439)	30,648 (431)	30,777 (429)	30,777 (429)	27,559 (425)
CHE	3887 (95)	2192 (127)	4167 (95)	4167 (95)	297 (63)

Acronyms as described in Table 1

DMI: dry matter intake; ECM: energy corrected milk; BW: body weight; ∆BW: change in body weight; DMI_RFI_: dry matter intake used to calculate RFI when ECM, BW, and ∆BW had no missing values

### Genotypes

The 824 Australian heifers were genotyped with the high-density (HD ~ 600k) SNP array. The genotypes of the 584 Australian cows were imputed to HD genotypes (~ 600k SNPs) using the Fimpute software [24]. The imputation from HD SNPs to WGS variants was performed using the Minimac3 algorithm [25]. The sequences of 3090 *Bos taurus* cattle representing multiple breeds and crosses from across the world (Run 7 of the 1000 Bull Genomes project, [26]) were used as reference animals for imputing from HD to WGS. Prior to imputation, the variants called in the cattle reference sequences were pre-filtered to retain only bi-allelic variants with allele counts of four or more (i.e. variants for which the alternate allele appeared less than 4 times were excluded), a Beagle *R*^*2*^ coefficient higher than 0.9, and a GATK Tranche score of 99.0 or more. In addition, variants with a heterozygosity higher than 0.5 were removed if they were located in chromosome segments (0.5 Mb) with an excessive heterozygosity higher than 0.5 (indicating alignment or mapping issues such as long tandem repeat regions) [27]. Minimac3 requires pre-phased genotypes in both the reference (WGS) and target sets, prephasing was done using the Eagle software [28].

The genotypes of the USA, CAN, DNK, and CHE cows were downloaded from the EDGP database, and the 50k genotypes of NLD and GBR cows were part of the dataset used in the development of the 2015 EBV for Feed Saved, as described by Pryce et al. [22]. The genotypes of cows from the different countries in the EDGP database were obtained from a variety of medium to HD chips, ranging in size from 55,647 to 777,961 SNPs.

Prior to imputation, all positions of the genotypes for each country were prepared according to the ARS-UDC1.2 sequence build of the bovine genome [29], and all unknown chromosomes and/or positions were removed. The allele frequency of each SNP across all countries was checked to ensure that the coding protocols for homozygotes were likely to be in the same direction (see Additional file 1: Figure S1). The missing genotypes for animals from each country were imputed separately up to the HD SNP array using a reference population of 2700 Australian animals that were genotyped directly with the HD SNP chips. Then, all HD genotypes were collated, and for consistency with the WGS data, were all converted to forward/forward sequence format and then were phased using the Eagle software. Imputation from HD to WGS was carried out as for the Australian animals described above. The genotypes on the *Bos taurus* (BTA) X chromosome were not included in the subsequent analyses.

### Population structure

The genomic relationship matrix (GRM) was constructed using HD genotypes of all 3711 animals from the seven countries using the method of Yang et al. [30]. Since the degree of relatedness between reference and validation populations has a strong impact on the accuracy of genomic prediction, we undertook several tests to better understand the relationships within and across datasets. First, a principal component analysis was performed on the GRM to show the degree of diversity among the 3711 genotypes of the animals from the seven countries. The numbers of animals per country used in this analysis are in Table 4. Also, a neighbour-joining (NJ) tree representing the genetic distance between each pair of countries’ groups of animals was drawn using the pairwise *F*_ST_ coefficients, which were calculated using the HD genotypes of all cows by adopting the formula of Hedrick [31]. In addition, the heterozygosity predicted from the GRM (*HE*_*pr*_) was compared to the mean observed heterozygosity (*HE*_o_) per country and the heterozygosity assuming Hardy–Weinberg equilibrium (*HE*_*oHW*_). *HE*_*pr*_ was calculated as: *HE*_base*_(1− *F*), where *F* is the inbreeding within each breed relative to the base population [8] and *HE*_*base*_ is the heterozygosity in the base population calculated as $\sum_{i}2{p}_{i}(1-{p}_{i})/N$, where ${p}_{i}$ is the allele frequency of the $i$th SNP across all datasets and $N$ is the total number of SNPs. The *HE*_o_ per country was calculated as the average number of heterozygous SNPs for each animal from each country.

### Statistical analyses

#### Genomic prediction

In total, 40,510 (50k) and 620,269 (HD) SNPs, which overlapped with WGS variants and were in common among the AUSc, AUSh, and OVE datasets, were used to build the 50k and HD GRM, respectively. Genomic prediction analyses were performed using two approaches:

1. either by using a multivariate model where the AUS cow and heifer datasets and the OVE dataset were analysed by treating them as correlated traits (bivariate or trivariate models), or using the combined AUS cow and OVE cow datasets by treating them as a single trait;
2. or by using the most significant variants selected from the WGS variants combined with either the 50k or HD SNP array to mimic a scenario where sharing of the raw data among countries is not feasible. The sequence variants were selected from the results of the GWAS, which was undertaken based on the OVE dataset, and were then used for the AUS cows to improve the genomic accuracy.

#### Validation and training populations

Only the AUSc dataset was used as validation population. Validation animals were split in two different ways: cohort-by-birth year and fourfold random cross-validations (where we avoided paternal half-sibs to be included in the reference and validation sets). For the cohort split, individuals were split into four sets by allocating animals according to their year of birth (2013, 2014, 2015, and 2016 + 2017 (in this case, a combined set of 2016 and 2017 animals was used due to the small sample size for these last 2 years). Any remaining animals were retained in the training sets. For the random split (fourfold cross-validation), any animals with unknown sires were retained in the training set (but not in the validation set) and the remaining individuals were split into four sets by allocating all offspring of randomly selected sires into one of the four datasets. Thus, the analysis was performed four times using each data fold in turn as a validation group and the other three folds used in the training population (i.e., 3-folds plus the remaining cows from above).

For the bi-variate model, the training populations were obtained by adding sequentially and in turn: the AUSh, EU, NA, and OVE cow datasets to the AUSc dataset (referred to as AUScAUSh, AUScEU, AUScNA, and AUScOVE, see Table 1). For the tri-variate model, the training populations were obtained by adding in turn the EU, NA, and OVE cow datasets to the AUScAUSh dataset (referred to as AUScAUShEU, AUScAUShNA, and AUScAUShOVE, see Table 1). For the univariate analysis in which the AUSc and OVE cow datasets were treated as a single-trait dataset, we used the same animals as in the AUScEU, AUScNA, and AUScOVE training populations.

#### Selecting sequence variants from GWAS for genomic prediction

##### Single-trait GWAS

To capture the associations between sequence genotypes and feed efficiency, we undertook a GWAS for both RFI and DMI. The single-trait GWAS for RFI and DMI were performed using the OVE dataset. The mixed model used for the GWAS fitted each sequence variant as a covariate, one at a time, and tested for an association with each trait:

$$\mathbf{y}={\boldsymbol{1}}_{\mathrm{n}}\upmu +\mathbf{X}\mathbf{b}+{\mathbf{s}}_{i}{{\upalpha }}_{i}+\mathbf{g}+\mathbf{e},$$

where $\mathbf{y}$ is the vector of observed phenotypic values of the animals, $\mathbf{1}_{\varvec{n}}$ is an $\mathrm{n}\times 1$ vector of 1s ($\mathrm{n}$ = number of animals with phenotypes), $\upmu$ is the overall mean, $\mathbf{X}$ is a design matrix relating observations to the corresponding fixed effect (dataset), $\mathbf{b}$ is a vector of fixed effects, ${\mathbf{s}}_{i}$ is a vector of genotypes (coded as 0, 1, and 2) for each animal at the $i$th variant, ${{\upalpha }}_{i}$ is the covariate effect of the corresponding variant, $\mathbf{g}$ is a vector of GEBV $\sim \mathrm{N}(\mathbf{0}, \mathbf{G}{\upsigma }_{\mathrm{g}}^{2}$), where ${\upsigma }_{\mathrm{g}}^{2}$ is the genetic variance and $\mathbf{G}$ is the GRM constructed from HD SNPs, and $\mathbf{e}$ is residual error. For a variant to be included in the GRM, its minor allele frequency had to be higher than 0.01, once the genotypes (real and imputed) were combined in the whole dataset. The analysis was performed using the GCTA software [32].

##### Multi-trait meta-analysis

A multi-trait, meta-analysis [33] was used to identify plausible individual and pleiotropic variants associated with feed efficiency. The multi-trait, meta GWAS (m-tr_G) was performed based on the signed-t values of estimated effects of sequence variants from the two single-trait GWAS (s-tr_G for DMI and RFI). The multi-trait ${\upchi }^{2}$ statistic for m-tr_G was calculated as in [33]:

$${\text{multi-trait }}{\upchi }^{2}={\mathbf{t}}_{\mathbf{i}}^{\mathbf{^{\prime}}}{\mathbf{V}}^{-1}{\mathbf{t}}_{\mathbf{i}},$$

where ${\mathbf{t}}_{\mathrm{i}}$ is a vector of the signed t-values of the effects of the $\mathrm{i}$-th SNP for DMI and RFI and ${\mathbf{V}}^{-1}$ is the inverse of the 2 × 2 correlation matrix where the correlation is calculated over all the estimated SNP effects (signed t-values) between DMI and RFI.

##### Selecting the most significant variants

In total, 31,380,025 WGS autosomal variants (excluding variants on BTA X) were used to perform the GWAS. Bolormaa et al. [34] showed that the Minimac3 *R*^*2*^ statistic is a good proxy for empirical imputation accuracy for use in filtering poorly imputed variants. According to their study, a Minimac3 *R*^2^ value greater than 0.4 corresponded to an empirical imputation accuracy of ≥ 0.87 (measured as the correlation between real and imputed genotypes). Approximately 60% of the 31,380,025 variants (18,921,317) had a Minimac *R*^2^ value greater than 0.4 in the OVE imputed WGS dataset (see Additional file 2: Table S1). About 96% of these 18,921,317 variants in the OVE cow dataset overlapped with the variants (*R*^2^ > 0.4) in the AUS cow WGS dataset. The sequence variants were removed if imputation *R*^2^ was less than or equal to 0.4 in both the OVE and AUS cow datasets, and variants also were removed if their minor allele frequency was lower than or equal to 0.006 in the OVE dataset. This resulted in 14.6 million sequence variants, which were available for further analysis. To avoid selecting a large number of closely-linked variants, only the three most significant variants with *P*-values < 0.001 were selected from within each 100-kb window along each chromosome, and sliding by 50 kb to the next window. This variant selection approach was undertaken using: (1) the results from single-trait GWAS (s-tr_G) for DMI and RFI, (2) the results from multi-trait, meta GWAS (m-tr_G), and (3) the combined variants from s-tr_G for RFI, s-tr_G for DMI, and m-tr_G (sm-tr_G).

#### GBLUP model

RFI was analysed as a single- and multi-trait genomic model based on restricted maximum likelihood (GREML) analyses with different combinations of datasets including AUSc, AUSh, and OVE cows. The analyses were performed using the ASReml package [32]. GEBV were calculated based on the following model:

1 $${\mathbf{y}}_{\mathbf{T}}={\mathbf{X}}_{\mathbf{T}}{\mathbf{b}}_{\mathbf{T}}+{\mathbf{Z}}_{\mathbf{T}}{\mathbf{g}}_{\mathbf{T}}+{\mathbf{e}}_{\mathbf{T}},$$

where ${\mathbf{y}}_{\mathbf{T}}$ is a $\mathrm{T}\times \mathrm{n}$ matrix of observations for $\mathrm{T}$ traits ($\mathrm{T}$ = 1, 2, or 3), ${\mathbf{X}}_{\mathbf{T}}$ is the incidence matrix for fixed effects, ${\mathbf{b}}_{\mathbf{T}}$ is the matrix of fixed effects (in this case, the mean for each trait and country of origin), ${\mathbf{Z}}_{\mathbf{T}}$ is an incidence matrix relating trait records to animals, ${\mathbf{g}}_{\mathbf{T}}$ comprises GEBV for T traits for animals with genotypes, distributed as $N(\mathbf{0}, \mathbf{G}\otimes \mathbf{P})$ where $\mathbf{P}$ is a $T\times \mathrm{T}$ matrix of additive genetic (co)variances between RFI of the AUSc, OVE cows, and/or AUSh, and $\mathbf{G}$ is the animal by animal GRM. ${\mathbf{e}}_{\mathbf{T}}$ is a $T\times \mathrm{n}$ matrix of residuals with $\mathrm{var}({\mathbf{e}}_{\mathbf{T}})=\mathbf{R}\otimes \mathbf{I}$, where $\mathbf{R}$ is a $T\times \mathrm{T}$ matrix of residual (co)variances and $\mathbf{I}$ is an $\mathrm{n}\times \mathrm{n}$ identity matrix. The GRM used in the GREML analysis was built based on the genotypes from either 50k or HD SNPs using the method of Yang et al. [30]. SNPs with a minor allele frequency higher than 0.01 to 0.05 (to ensure that a minimum of 20 alleles per variant were segregating in each of the reference populations) were included in the GRM.

For scenarios for which the most significant variants from the WGS GWAS combined with the 50k SNP array were used in the GREML analysis, first the GRM were built separately using either 50k SNP genotypes (GRM_SNP_) or genotypes for the selected sequence variants (GRM_seq_) in each set. Then, the GREML analyses were performed using the phenotypes of the reference populations to estimate the genetic variance explained by the GRM_SNP_ (σ^2^_SNP_) and GRM_seq_ (σ^2^_seq_). The genetic variance estimates were used as weights to aggregate the GRM_SNP_ and GRM_seq_ into a single weighted GRM (GRM_weighted_): GRM_weighted_ = (σ^2^_SNP_ * GRM_SNP_ + σ^2^_seq_ * GRM_seq_)/(σ^2^_SNP_ + σ^2^_seq_) as described by Khansfield et al. [35]. The weighted GRM was then used for the GBLUP analyses to estimate the GEBV in the validation population. The accuracy of the predicted GEBV was compared to the accuracy of GEBV predicted by using only the 50k or HD SNP GRM.

#### Accuracy of GEBV

For each validation population (AUSc), the accuracy of genomic prediction was calculated as the correlation between GEBV and the phenotype corrected for fixed effects. Then, the correlation was divided by the square root of the genomic heritability of the trait (*h*^*2*^) in the AUSc dataset. The *h*^*2*^ was estimated as the proportion of the phenotypic variance that was explained by the 50k SNPs. Estimates of genomic heritability and of the accuracy of GEBV were averaged across the four validation sets for each trait. The standard error of the accuracy of GEBV was estimated from the four randomly sampled independent fold validation sets for each trait (i.e., as the standard deviation of the 4 accuracies divided by the square root of 4). Note that this is an approximate standard error because the reference fold sets are not completely independent from each other.

## Results

### Population structure

**Fig. 1:**
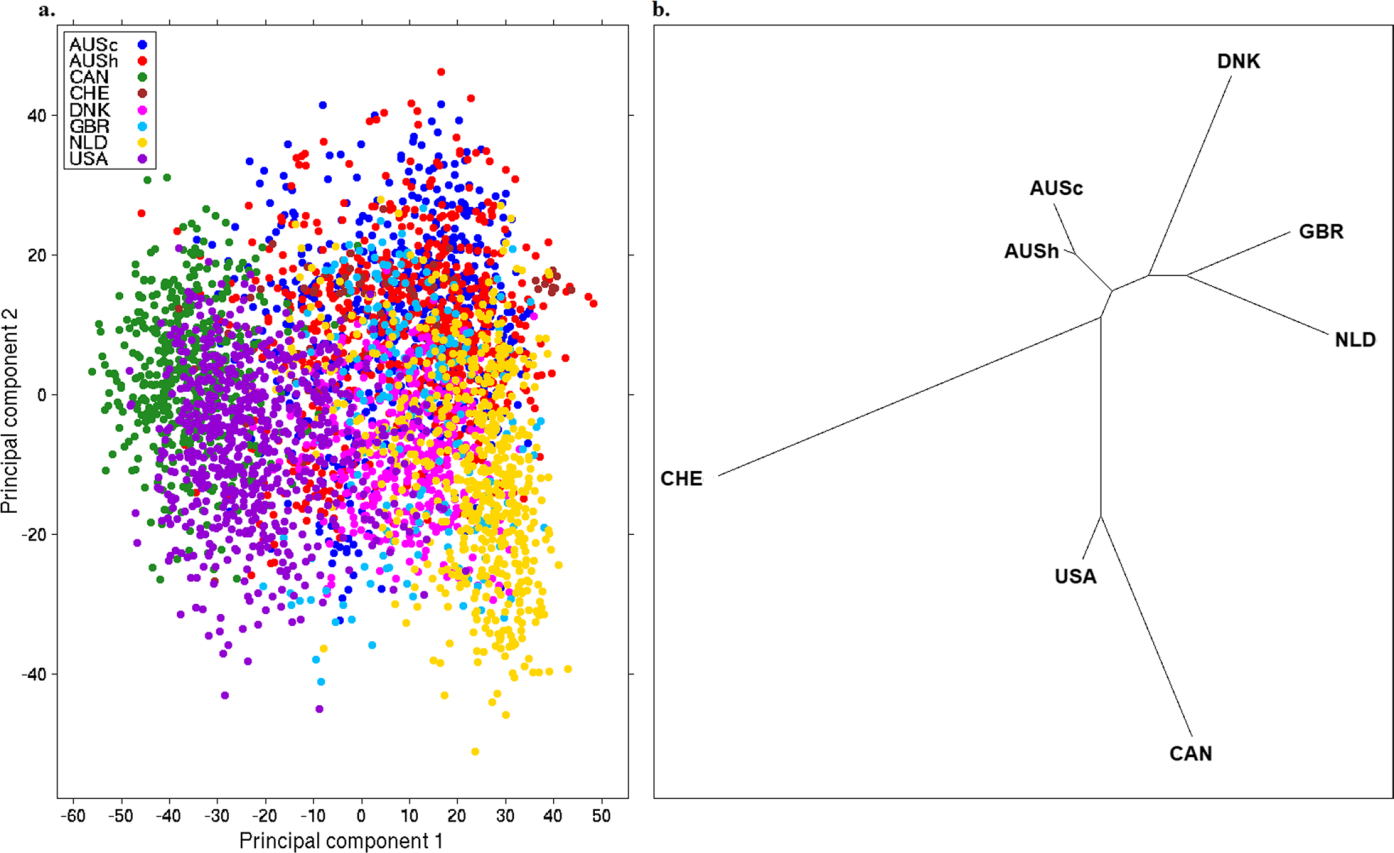
**a** Principal component decompositions of the genomic relationship matrix constructed from HD SNP genotypes for 3711 animals from seven different countries and **b** Neighbor-Joining tree representing the genetic distances between animals from different countries

The first two principal components from the GRM were used to show the degree of differentiation between the genotypes of the 3711 cows from the seven countries. As expected, all cows (Holsteins) appeared as a single cloud (one breed) (Fig. 1a). The USA and CAN cows were slightly separated from the others, as shown on the left side of the first principal component (PC1) axis, while the remaining groups were either in the middle or on the right side of the PC1 axis. This separation is clearly shown on the NJ tree in Fig. 1b, which displays four main clusters: USA and CAN cows formed a separate cluster on the opposite side of all other clusters. As expected, AUS cows and heifers belonged to the same cluster, and the European countries of DNK, GBR, and NLD also formed their own cluster, while CHE sat outside this cluster.

There was agreement among these population measures, with all the genotype groups displaying a similar range of heterozygosity (0.32–0.34) (Table 3), which shows that the GRM constructed with animals from different groups is a reliable representation of the relationships among and within groups of animals.

**Table 3:** Observed and predicted heterozygosities for each genotype set

Dataset	Number of animals	HEpr	HEo	HEoHW
AUSc	584	0.33	0.34	0.33
AUSh	687	0.33	0.34	0.34
USA	671	0.33	0.33	0.32
CAN	473	0.32	0.32	0.32
DNK	425	0.33	0.33	0.33
CHE	63	0.32	0.33	0.34
GBR	211	0.33	0.34	0.33
NLD	597	0.33	0.33	0.33

Acronyms as described in Table 1

Number of animals: number of animals used to calculate residual feed intake (RFI); HE_pr_: heterozygosity predicted from the GRM; HE_o_: observed heterozygosity; HE_oHW_: heterozygosity when SNP are assumed to be in Hardy–Weinberg equilibrium

### Genetic parameters

Stage of lactation affects DMI and, hence, we present the mean and standard deviation (SD) for DIM and DMI in the same table (Table 4). The mean DIM was lowest for the AUSc and USA cows and highest for the DNK cows. The experimental design resulted in a DIM for AUSc that spanned 32–245 DIM, however 67% of animals were recorded with the relevant traits between 75 and 124 DIM. Although the DIM for cows from other countries was restricted to be between 5 and 306 days, the mean and SD of DIM for the USA cows was smaller compared to those for the cows from other countries because about 78% of the DMI for the USA cows were recorded between 6 and 99 DIM. The SD of raw DMI was smaller for the CHE, AUSc, and DNK datasets compared with USA, CAN and GBR datasets. The SD of DMI corrected for fixed effects was within a similar range (~ 2.6) for the AUSc, EU, NA, and OVE datasets. For RFI, the SD ranged from 1.17 to 2.26, except for the CAN cows, for which the SD was equal to 3.0. As expected, compared to the RFI for cows, the RFI for heifers had a lower mean and SD (Table 4).

**Table 4:** Mean of days in milk (DIM) (and standard deviation in parenthesis) and mean and standard deviation of raw and corrected dry matter intake (DMI) and residual feed intake per country and dataset

Dataset	Raw DMI			DMI^a^			RFI^a^		
	DIM	Mean	SD	Number	Mean	SD	Number	Mean	SD
AUSh		8.29	1.34	824	− 0.01	0.84	824	− 0.02	0.42
AUSc	108 (35)	22.93	3.80	584	− 0.02	2.10	584	0.00	1.29
USA	81 (67)	21.52	4.47	673	0.09	1.97	671	0.01	1.46
CAN	121 (77)	20.54	5.47	755	0.04	3.20	473	− 0.07	3.04
DNK	140 (83)	21.93	3.57	439	0.34	1.87	425	0.12	1.17
CHE	120 (79)	21.48	3.62	95	0.10	2.88	63	0.09	1.20
GBR	143 (83)	16.70	5.25	564	− 0.31	2.85	211	− 0.14	2.26
NLD	na	na	na	na	na	na	597	0.00	0.97
EU	na	na	na	1098	− 0.02	2.52	1296	0.02	1.33
NA	na	na	na	1428	0.07	2.69	1144	− 0.02	2.25
OVE	na	na	na	2526	0.03	2.62	2440	0.00	1.82

Acronyms as described in Tables 1 and 2

na: not applicable

^a^Corrected phenotypes for dry matter intake (DMI) and residual feed intake (RFI) in GBLUP

The *h*^*2*^ for DMI and RFI estimated with a univariate model in the different datasets that were used for genomic prediction analyses are in Table 5. DMI was moderately heritable, with an *h*^*2*^ ranging from 0.29 (EU) to 0.40 (NA). The *h*^*2*^ for RFI was equal to 0.19 for AUSc, 0.36 for AUSh, and ranged from 0.27 to 0.32 for the three combined datasets of international cows (EU, NA or OVE). The *h*^*2*^ estimates using the bivariate and trivariate models were comparable. The standard errors (SE) of *h*^2^ ranged from 0.032 to 0.090. The r_g_ from the trivariate analyses in which the three datasets (i.e., AUSh, AUSc, and OVE) were treated as correlated traits are in Table 6. For the AUScAUShOVE dataset, the r_g_ for RFI was 0.30 and 0.95 between AUSc and AUSh and between AUSc and OVE cows, respectively. As expected, the r_g_ of AUSh with OVE cows was comparably lower than with AUSc. The estimates of the r_g_ were associated with quite large SE (0.18–0.39).

**Table 5:** Number of animals, variance explained by 50k genotypes and residuals, and genomic heritability estimates for DMI and RFI within each dataset

Dataset	RFI				DMI			
	Number of animals	V_g_	V_e_	h^2^ (s.e.)	Number of animals	V_g_	V_e_	h^2^ (s.e.)
AUSh	824	0.37	0.65	0.36 (0.087)	824	0.37	0.65	0.36 (0.086)
AUSc	584	0.19	0.82	0.19 (0.088)	584	0.33	0.67	0.33 (0.090)
EU	1296	0.29	0.60	0.32 (0.053)	1098	0.23	0.55	0.29 (0.053)
NA	1144	0.26	0.71	0.27 (0.051)	1428	0.34	0.52	0.40 (0.045)
OVE	2440	0.26	0.68	0.27 (0.034)	2526	0.25	0.58	0.30 (0.032)
AUScEU	1880	0.28	0.65	0.30 (0.043)	1682	0.25	0.61	0.29 (0.044)
AUScNA	1728	0.23	0.75	0.23 (0.041)	2012	0.32	0.58	0.36 (0.038)
AUScOVE	3024	0.24	0.70	0.26 (0.030)	3110	0.25	0.61	0.29 (0.029)

Acronyms as described in Tables 1 and 2

**Table 6:** Genetic correlations (SE) for RFI in trivariate model using different scenarios

Dataset	Number of animals	AUSc-AUSh	AUSc-EU/NA/OVE	AUSh-EU/NA/OVE
AUScAUShEU	2567	0.30 (0.26)	0.68 (0.31)	0.28 (0.22)
AUScAUShNA	2415	0.37 (0.27)	0.90 (0.39)	0.19 (0.24)
AUScAUShOVE	3711	0.34 (0.26)	0.95 (0.28)	0.25 (0.18)

Acronyms as described in Tables 1 and 2

SE: standard error

### Genomic prediction

#### Using datasets treated as different traits

**Fig. 2:**
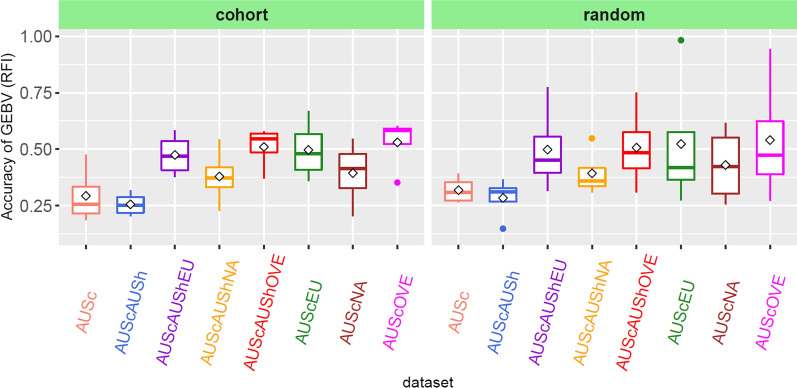
Box plot showing accuracies of GEBV for RFI using single- and multi-variate GREML analyses using cohort and random cross-fold validation approaches. As a training population, AUSc (Australian cows) used for the single-variate analysis; AUScAUSh, AUScEU, AUScNA, and AUScOVE (AUSc with Australian heifers (AUSh), European cows (EU), North American cows (NA), and overseas cows (OVE), respectively) used for the bi-variate analyses. AUScAUShEU, AUScAUShNA, and AUScAUShOVE (EU, NA, and OVE cow datasets on top of the AUScAUSh dataset) used for the tri-variate analyses

Figure 2 shows the genomic prediction accuracies for RFI obtained in the different scenarios (i.e., where we used different prediction models including univariate, bivariate, and trivariate analyses) with the 50k GRM. As expected, the accuracies estimated using the 50k GRM and HD GRM were nearly at the same level (not shown). The same validation population (i.e., AUSc) was used across scenarios. There were 118 cows (SD = 23) for each of the cohort-split datasets and 115 cows (SD = 6) in each of the random-split validation populations. In many cases, as expected, the accuracy estimated using the random split was slightly higher than that estimated using the cohort-split approach (Fig. 2). The overall pattern of accuracy across scenarios was the same regardless of whether the cohort- or the random cross-fold approach was used. Compared to the mean accuracy estimated using the univariate model, for which only AUSc were present in the training set, the accuracies improved when using bivariate and trivariate models, except when the AUScAUSh training population (i.e., where the AUS cows and growing heifers were combined) was used. The largest improvement in prediction accuracy was obtained when all OVE cows were included in the training datasets (i.e. AUScOVE and AUScAUShOVE) (Fig. 2). The training population that consisted of EU cows provided a greater accuracy for RFI compared to that of NA cows. Including AUSh in the training population (AUScAUShOVE) did not improve the accuracy for RFI compared to AUScOVE (Fig. 2).

### Using datasets treated as the same trait

**Fig. 3:**
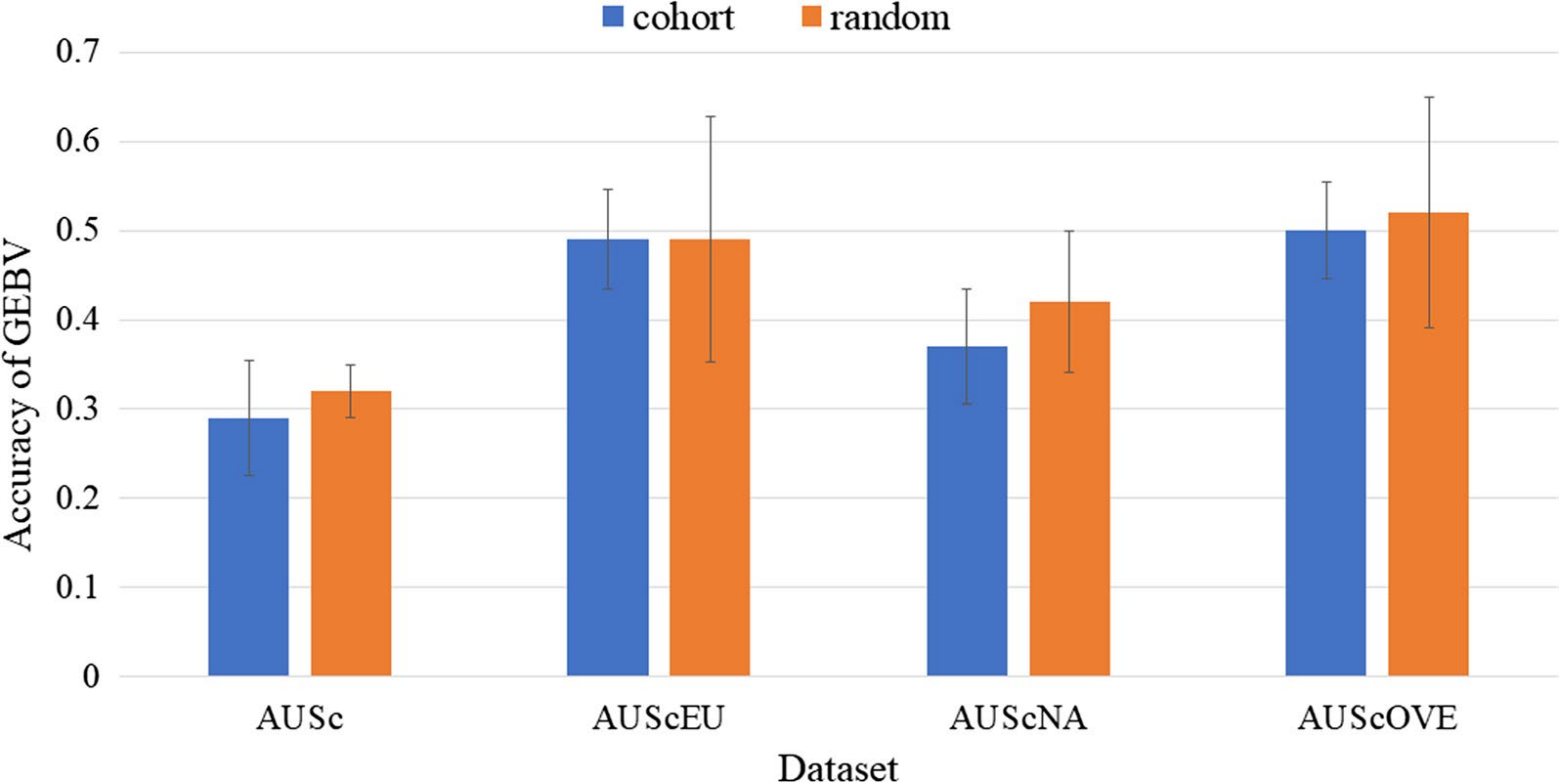
Mean accuracies of GEBV for RFI by treating Australian cow and overseas cow datasets as a single trait and using cohort and random cross-fold validation approaches. The standard error (SE) bars are approximate estimates. As a training population, AUSc (Australian cows) used for the single-variate analysis; AUScEU, AUScNA, and AUScOVE (AUSc with European cows (EU), North American cows (NA), and overseas cows (OVE), respectively) used for the bi-variate analyses

Because the r_g_ for RFI between AUSc and OVE cows were quite high (Table 6) and there were no overlapping animals with phenotypes, we merged the AUSc and OVE cow datasets so that the RFI phenotypes were treated as the same trait (Fig. 3). By combining the training populations AUScEU, AUScNA, and AUScOVE, the mean accuracies were around 0.4 to 0.5, which was 8 to 22% higher than the accuracy obtained by using only AUSc in the training set (Fig. 3). The improvement in accuracy was smallest when using the AUScNA cows as the training set.

### Using top sequence variants

#### WGS GWAS

To determine the relevance of using the variants selected from the GWAS, we used the largest dataset (OVE) as the discovery population. The number of animals used in the GWAS was 2440 for RFI and 2596 for DMI (Table 3). The overall power of single- and multi-trait, meta WGS GWAS was weak due to the small size of the datasets (see Additional file 3 Figure S2). For example, the largest number of significant sequence variants at *P* < 5 × 10^–5^ was 1690 in the multi-trait GWAS. Because the primary purpose of this study was focused on improving genomic prediction using sequence variants, we applied a rather lenient threshold *P*-value (*P* < 10^–3^) to select the most significant three variants (*P* < 10^–3^) within every 100-kb window, with a sliding window of 50 kb to generate a subset of sequence variants for genomic prediction. We selected four different sets of the ‘top sequence variants’ from the GWAS results: single trait GWAS (s-tr_G, number of variants (N) = 3359 for RFI and N = 3432 for DMI), multi-trait GWAS (m-tr_G, N = 4999), and combined set of s-tr_G and m-tr_G (sm-tr_G, N = 8946). These top (non-50k) sequence variants across the bovine genome were used in GBLUP in addition to the 50k SNPs.

#### Accuracy of GEBV

Table 7 shows the mean accuracies across different analyses and the slopes of the regression of corrected phenotypes on GEBV. A single-weighted GRM was tested using only the AUSc training and validation populations, where the GRM was built from 50k SNPs and top sequence variants that were weighted by the genetic variance explained by the selected sequence variants, as described in Khansefid et al. [35]. The accuracies of GEBV across the AUSc validation population using the weighted GRM with 50k SNPs + ‘top sequence variants’ selected from the OVE GWAS are comparable to the accuracies using GRM with only 50k or HD SNPs (Table 7). The proportion of the average weights of the variance explained by the top sequence variants against the variance explained by 50k SNPs ranged from 0.42 to 0.96 across different validation sets and splits. Consistent apparent improvements in accuracy were observed as the number of informative sequence variants increased (s-tr_G, m-tr_G, and then sm-tr_G). Across the different selected sequence variant scenarios, the general pattern of accuracies using cohort- and random-validation splits were very similar.

**Table 7:** Average weighted accuracies of GEBV for RFI and regression slopes of corrected phenotypes on GEBV (bias) of the four cohort and random fourfold cross-validation subsets

Training set	Number of traits^a^	Variant set	Accuracy	Bias	Difference^b^
Cohort					
AUSc	1	50k	0.29	1.13	
AUSc	1	HD	0.29	1.10	0.0
AUScAUSh	2	50k	0.25	0.90	− 3.5
AUScOVE	2	50k	0.52	1.31	22.9
AUScAUShOVE	3	50k	0.50	1.28	21.1
AUScOVE	1	50k	0.50	0.99	21.8
AUSc	1	50k + s-tr_G	0.34	1.28	5.6
AUSc	1	50k + m-tr_G	0.38	1.10	9.0
AUSc	1	50k + sm-tr_G	0.39	1.15	10.8
Random					
AUSc	1	50k	0.32	1.07	
AUSc	1	HD	0.32	1.14	0.5
AUScAUSh	2	50k	0.29	0.84	− 3.0
AUScOVE	2	50k	0.53	1.26	21.3
AUScAUShOVE	3	50k	0.50	1.17	18.4
AUScOVE	1	50k	0.52	0.93	20.1
AUSc	1	50k + s-tr_G	0.34	1.17	1.8
AUSc	1	50k + m-tr_G	0.37	1.10	5.5
AUSc	1	50k + sm-tr_G	0.38	1.15	6.6

Acronyms as described in Table 1

^a^Number of traits (datasets) used in each analysis

^b^Difference in accuracy of GEBV (%) between using the Australian cow dataset (AUSc) with 50k GRM and the corresponding dataset

As shown in Table 7, the accuracies using only 50k and HD SNPs were almost the same. Compared to using only 50k or HD SNPs, accuracies improved by up to 22% for RFI when using international data and either treating traits from international data as independent traits or merging them as single-trait or top sequence variants from GWAS with international datasets. Bivariate (AUScOVE) and trivariate analyses (AUScAUShOVE) yielded the highest accuracies that were within a similar range. The accuracies of genomic prediction estimated by using selected sequence variants with 50k SNPs were lower compared to the accuracies estimated using the individual cow phenotypes (AUSc + OVE datasets). Accuracy increased as the number of informative sequence variants increased (Table 7). The mean slopes of the regression of corrected phenotypes on GEBV across validation sets were closer to 1.

## Discussion

This study shows that in a small cross-validation study, the inclusion of additional data on feed efficiency traits from international partners increases the accuracy of genomic prediction of breeding values. Although collating datasets from additional sources (e.g., international cow data) is crucial to improve the accuracy of expensive-to-measure traits, there are two ways in which the information can be used to improve the accuracy of genomic prediction: (1) sharing of phenotypes and genotypes, or (2) sharing of signed t-values of SNP effects only to generate a custom set of more highly predictive variants. Both of these strategies were effective for increasing the genomic prediction of RFI although higher accuracies were achieved from sharing the full data.

### Genetic parameters

The *h*^2^ estimates were moderate, ranging from 0.32 to 0.40 for DMI and from 0.20 to 0.36 for RFI. The smaller SE of the OVE *h*^2^ estimate of RFI compared to that of the AUSc reflects the larger number of cows used in the OVE dataset. The estimates obtained in the AUSc dataset were the same as those previously published by Pryce et al. [22], which is not surprising given that the 235 cows in that study were part of our dataset. Other studies have reported various estimates for the *h*^*2*^ of RFI and DMI in lactating cows, ranging from 0.12 [36, 37] to 0.38 [38] and from 0.20 [39] to 0.40 [40], respectively. The *h*^2^ estimate for RFI in 417 growing heifers reported by Korver et al. [41] was 0.22, which is lower than that obtained in our study on 824 heifers.

The *h*^2^ and r_g_ estimated with the bivariate and trivariate models where the AUSc, AUSh, and OVE cow datasets were treated as individual traits were all very similar. The limitation of using a multivariate analysis was the small dataset and thus the resulting large SE associated with the genetic parameters, particularly for the r_g_ of traits in AUSc with the equivalent trait in other countries. For example, the r_g_ (± SE) of RFI between AUSc and OVE cows was 0.95 (± 0.28) and between AUSc and EU cows was 0.68 (± 0.31), which are higher than the estimates of 0.60 reported by Pryce et al. [22], but with a very large SE (± 0.60). The r_g_ of RFI between growing heifers and lactating cows was much lower. In fact, the highest r_g_ (0.37 ± 27) was found for AUScAUShNA, which was considerably lower than those reported by Pryce et al. [22] (0.67 ± 0.45) and Nieuwhof et al. [42] (0.74 ± na) using Dutch lactating cows and heifers but again with quite large SE. The r_g_ of DMI between AUSc and OVE cows ranged from 0.64 (± 0.28) to 0.82 (± 0.24), which are within the estimates reported in different populations (0.14–0.84) and closer to the estimates of 0.76–0.84 in the high input European production system [15].

### Genomic predictions

The accuracies of GEBV estimated in the different scenarios tested in this study ranged from 0.25 to 0.53 for RFI and increased by up to 21% when international data (OVE cows) were added to the AUSc or AUScAUSh datasets. Therefore, such collaborations are beneficial to genomic prediction. A steep increase in the accuracy of GEBV for RFI (up to 21%) was observed as the size of the training population increased as expected from the theory [43] that traits with larger training populations and/or high *h*^2^ achieve higher accuracies of GEBV. Some studies in cattle and sheep [8, 17, 44] reported that using an enlarged mixed breed training population leads to higher accuracies for breeds with small training populations.

In this study, we used data that were collected during the lactation period (DIM: 5–306 days). The traits used were measured at different stages of lactation and in different environments. The duration of the experiments in USA and CAN were longer and therefore, the number of records per cow in these studies was much larger than that of the AUSc dataset, for which most of the records (~ 70%) were deliberately collected between 75–124 DIM. To capture the effect of lactation period, we used the approach of Berry et al. [15] that divides the lactation into four stages and then combined lactation stage with the parity levels for OVE cows.

While all the cows used in this study were Holsteins, they were from different countries and continents. The populations from North America and Europe were managed under comparatively higher input systems (i.e., fed more concentrates) than the Australian cows, that were fed a diet predominantly of lucerne cubes with some concentrates fed at milking time. A possible genotype-by-environment interaction effect could exist across broader groups of cows (across continents). To address this issue, we used three training sets (NA, EU, and OVE cows in addition to AUSc or AUScAUSh). However, even within these populations, sub-populations seem to be present. The PC plot obtained from the GRM and the NJ tree constructed using *F*_ST_ coefficients, which were calculated using HD genotypes, showed that the CHE cows are separated from other EU cows. However, the *HE*_*o*_ and *HE*_*pr*_ estimates for the CHE cows were in the same range, with no distortion in the within-population genetic variance (Table 3). We always fitted dataset as a fixed effect to account for population differences, so the relationships between populations were not used in the calculation of GEBV and in the GWAS. Therefore, we assumed that the GRM, which was constructed from different populations and used for genomic prediction, correctly reflected the amount of genetic variation within each population. In some cases, a distortion in the within-population (breed) genetic variance could occur. This is especially the case when collating data from different breeds (Holstein and Jersey in dairy cattle or different breeds in beef cattle and sheep). For example, Erbe et al. [45] reported such a distortion in the data collated on a large sample size from the Holstein dataset and a small sample size from the Jersey dataset.

We also observed some differences in the variance estimates of the studied traits (see Table 4). Compared to the AUSc population, a slightly greater standard deviation in RFI was found for the NA cows. Such differences can be appropriately quantified or accounted for in the multivariate analysis by using the (co)variances between the variables (traits), and hence a multivariate analysis could be a better option than a single-trait model when all the cows' phenotypes are collated. However, the multivariate analyses (that treat the AUSc and OVE cow datasets as correlated traits) used in this study yielded very similar levels of accuracy to those of the single-trait analyses of AUSc combined with OVE (Table 7).

The accuracies of genomic prediction for RFI using the training set were comparatively higher when it was supplemented with EU cows than with NA cows. It is not surprising that, compared to the NA cows, the size of the training populations for RFI was slightly larger for the EU cows and variation of RFI in AUSc and EU were at similar levels. A few studies have investigated the accuracy of genomic prediction estimates for feed intake and efficiency, particularly in lactating cows (e.g. [7, 10]). Based on a dataset of 1801 Holstein lactating cows and heifers from three research herds in Australia and Europe, Haas et al. [7] reported that the estimated accuracy of genomic prediction for DMI ranged from 0.33 to 0.39 using a multivariate (3-country) model. In the most recent report by Li et al. [46], the average theoretical reliability was estimated to be 0.34 in 3.9k Holstein cows with RFI phenotypes and genotyped for 61k SNPs.

The accuracies of GEBV using the GRM constructed from 50k SNP genotypes were at almost the same level as those using a GRM from HD SNP genotypes, probably because of long-range LD sharing across Holstein animals and hence, the 50k SNP density was as effective in tagging QTL as the HD SNP. In addition to the 50k SNPs, the variants from imputed sequence data selected in the discovery set (independent, international cow data) increased the accuracy of genomic prediction in the AUSc dataset. Several other research groups also reported an increase in genomic prediction accuracies by combining more predictive variants from imputed sequence data with a standard SNP chip, particularly for across-breed prediction, or prediction of animals that are weakly related to the reference animals [17–21, 47, 48].

Causal sequence variants may be rare and, hence, in low LD with the SNPs on the SNP arrays. On the one hand, the study by Druet et al. [49] who used WGS variants for genomic prediction showed that, compared to dense SNP arrays, the greatest improvement in accuracy was reached when causal variants were rare. On the other hand, in a within-breed study, Veerkamp et al. [50] reported no advantage in using selected sequence variants compared to the standard 50k SNP genotypes for several traits in dairy bulls. However, compared to our study, they used a larger reference population with accurate breeding values for bulls and a validation set that appeared to be strongly related to the reference set. These key differences resulted in reasonably high base accuracies of genomic prediction for the traits studied, and the results reported in [18, 36] demonstrated that in such a setting, further improvements in accuracy are more difficult to achieve. In addition, Veerkamp et al. [50] used a sequence variant discovery set that was not independent from the reference set (i.e. the discovery and reference sets were the same population). It has been shown that the use of an independent discovery set can lead to improved accuracy and less bias compared to when the reference and discovery population are not independent [18]. The power of GWAS is generally inadequate to detect variants that explain all the genetic variance in highly polygenic traits (e.g., [30]), and hence it is important to combine selected sequence variants with the standard 50k SNP array (using a weighted approach). In addition, we pre-filtered the imputed sequence variants (Minimac3 *R*^*2*^ > 0.4) for imputation quality and applied a MAF threshold (MAF ~ 0.01), which are important factors to reduce the level of false positives in the GWAS. In our study, the external independent data (international data) were used to select the sequence variants, which were subsequently used in genomic prediction for the Australian cows. The genetic variation for DMI varies across lactation stages and the genetic correlations between DMI at different stages of lactation were lower than 1 [36]. Several studies [36, 51–53] reported that the heritability for RFI varies with the stage of lactation. Some overseas data recorded DMI for a range of lactation stages (early to late) or across the entire lactation (e.g., DNK) and this possibly increased the power to identify the sequence variants that affect feed efficiency traits.

In our study, the relative increase in accuracies obtained by using the selected sequence variants with 50k SNPs were lower than the accuracies estimated using the external phenotypes (OVE) with the AUSc dataset, but were consistently higher than when only 50k or HD SNP genotypes were used. The increase in accuracy was generally largest for the SNPs that were selected and combined from the single and multi-trait meta-GWAS, as expected, because the multi-trait GWAS increases the power to detect pleiotropic variants [33], while the single trait meta-GWAS is likely to complement the selection with variants that are not pleiotropic and individually do not have a large effect. Although in theory the QTL for the DMI component of RFI can be detected in the RFI GWAS, the multi-trait GWAS may help to amplify the QTL with smaller effects that might have been otherwise missed given the small size of the data sets. While our results should be interpreted with caution due to the small size of the reference and validation sets used in this study, it is possible that the apparent success was also in part due to the OVE discovery set being five times larger than the training population. If using selected sequence variants in addition to the 50k SNPs proves to be an advantage, it could be a great opportunity with each contributing country providing GWAS results (signed-t values) for a meta-analysis to generate custom SNP sets instead of sharing raw individual cow data. Indeed, combining raw datasets from different institutes is a very time-consuming and tedious work that is fraught with possible errors. It is unlikely that individual countries on their own can generate large enough RFI phenotype datasets to achieve high genomic prediction accuracy. Therefore, it is crucial to maintain international collaboration to provide access to more data or to combine GWAS results from more countries. The latter may be more straightforward to implement in practice. However, the disadvantage of using GWAS data is that there would be a lag-period before the industry benefits from them because it is necessary to design custom genotyping platforms that the commercial (dairy) industry can use to genotype or impute these SNPs. In addition, currently the cost is often associated with increasing the number of SNPs on genotyping platforms.

We used two validation approaches, i.e. one using cohort-by-birth year sets treated as different validation sets and the other using random cross-validation sets, which, whenever possible, do not allocate the same sires’ offspring to the training and validation sets. In most cases, the relative increase in accuracy of GEBV using random validations was slightly greater than using the cohort validations. This is probably because animals in the training and random validation sets are more closely-related than those in the cohort-split datasets. A strong relationship between the animals in the training and validation populations is likely to lead to a greater increase in accuracy due to the sharing of long haplotypes as observed previously in cattle and sheep [18, 54]. Another consideration is that the size of the validation population used in this study was small. Therefore, it is necessary to continue these studies with larger training and validation populations to confirm if our findings can be generalised for other traits and breeds. Although the achieved accuracies of GEBV for feed intake and efficiency were lower than those for production traits, they are sufficient for implementation in commercial breeding programs.

The improvement of the genomic prediction accuracy obtained for feed efficiency using sequence variants is interesting since it is an expensive and difficult-to-measure trait with limited data availability. Improving the precision of variant detection not only contributes to better understand the underlying biology, it can also be valuable to enhance and improve the accuracy of genomic prediction, as shown here. Adding AUSc data to the international dataset increased the power for identifying the putative causal sequence variants, but the main focus of our study was to implement genomic prediction using international cows as an independent discovery population and to select the top sequence variants for improving the accuracy of genomic prediction. Furthermore, integrating feed efficiency data with other phenotypic sources, such as ruminal microbes and potential intermediate phenotypes (e.g., metabolites and milk mid-infrared spectroscopy) could help identify potential physiological mechanisms to enhance feed uptake and production efficiency. In a study on a Holstein cattle population, Delgado et al. [55] showed that there is an association between the rumen microbiota and traits related to feed efficiency using whole metagenome sequencing (e.g., cows with a greater relative abundance of *Bacteroidetes* were more efficient at feed utilization.). A few other studies [56, 57] have also shown that certain rumen microbial features are heritable and that their abundance is significantly influenced by the host genetics.

## Conclusions

The accuracy of genomic prediction of RFI improved when more than 3700 Australian cows and heifers and overseas cows were used by fitting a multivariate model. The results suggest that the accuracies of feed efficiency will improve if the training population is increased in size through international collaboration. The predictive sequence variants from the meta-GWAS of overseas data combined with the 50k SNP array data also provided an apparent increase in accuracy, but it was smaller than that obtained by combining the phenotypic datasets. However, this result should be interpreted with caution because of the small size of the reference and validation sets used in this study, and repeating the approach with larger datasets may be informative. However, our finding is important, because when international data are limited by sample size, sharing genetic variants from a GWAS approach is likely to be an effective alternative to sharing phenotype and genotype datasets. Since the current training population based on Australian animals remains small, international collaboration is crucial to achieve more accurate EBV for feed efficiency in Australia. The standard errors of genomic parameters, including estimates of heritability and genetic correlations, were large and hence, further studies with larger datasets are required to obtain more accurate estimates.

## Supplementary Information

**Additional file 1: Figure S1.** Allele frequency of HD SNPs in Australian cows (on the X axis) and Australian heifers or cows from each international country.

**Additional file 2: Table S1.** Number of filtered polymorphic variants (Minimac *R*^*2*^ > 0.4) per chromosome in the imputed WGS for the overseas cows.

**Additional file 3: Figure S2.** –log_10_(*P*-values) of single SNP regressions from single-trait GWAS for residual feed intake (RFI, a) and dry matter intake (DMI, b), and multi-trait meta GWAS (mt-tr, c) using the overseas (OVE) cow dataset. The orange points represent selected significant variants at *P* < 10^–3^.
