# Supplementary figures and images for: Sharing of either phenotypes or genetic variants can increase the accuracy of genomic prediction of feed efficiency
Source: Genet Sel Evol. 2022 Sep 6;54:60. doi: 10.1186/s12711-022-00749-z (PMC9450441; doi:10.1186/s12711-022-00749-z)

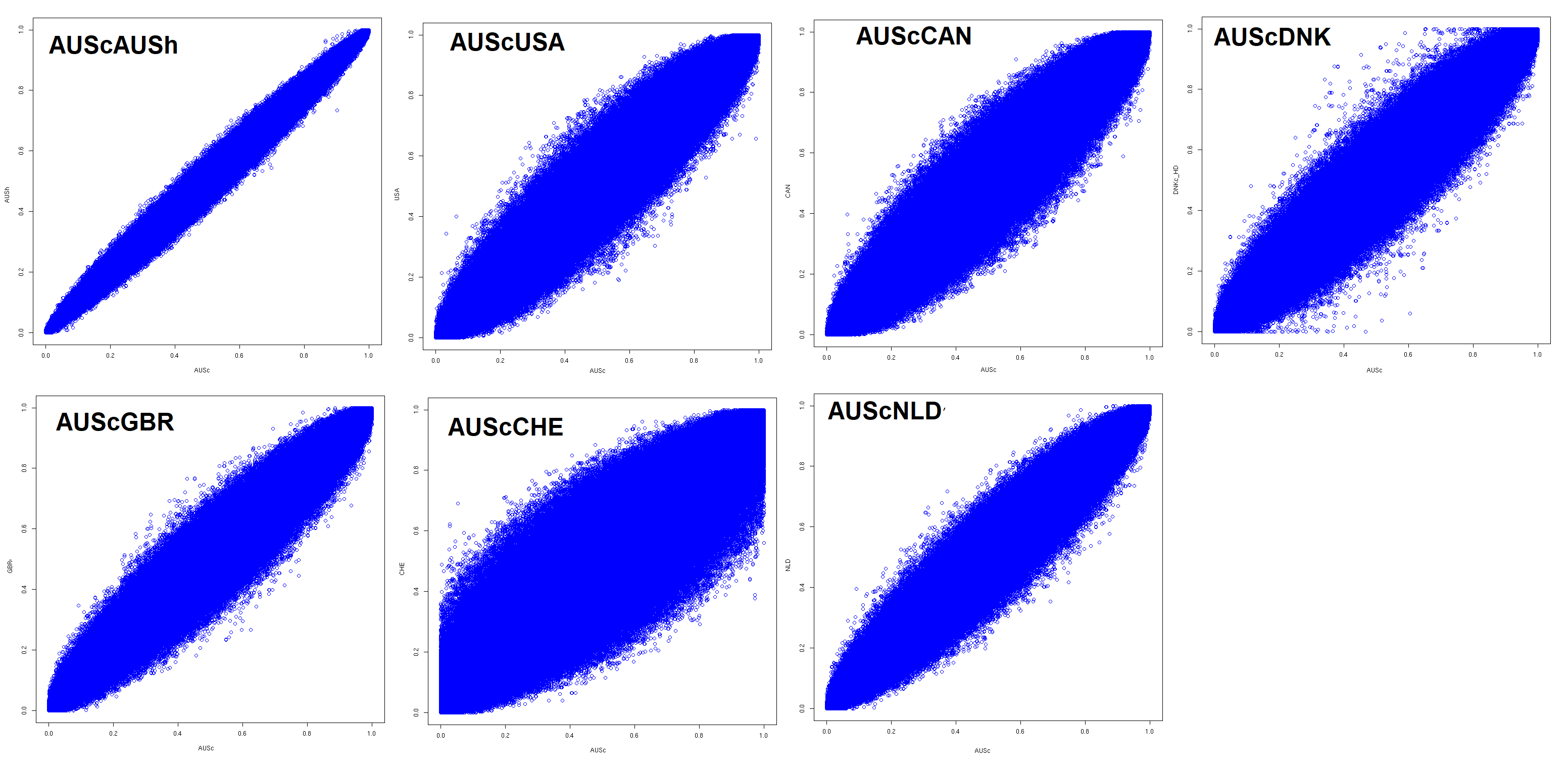

Supplement: Supplementary file 1 — Additional file 1: Figure S1. Allele frequency of HD SNPs in Australian cows (on the X axis) and Australian heifers or cows from each international country. [file 12711_2022_749_MOESM1_ESM.png]

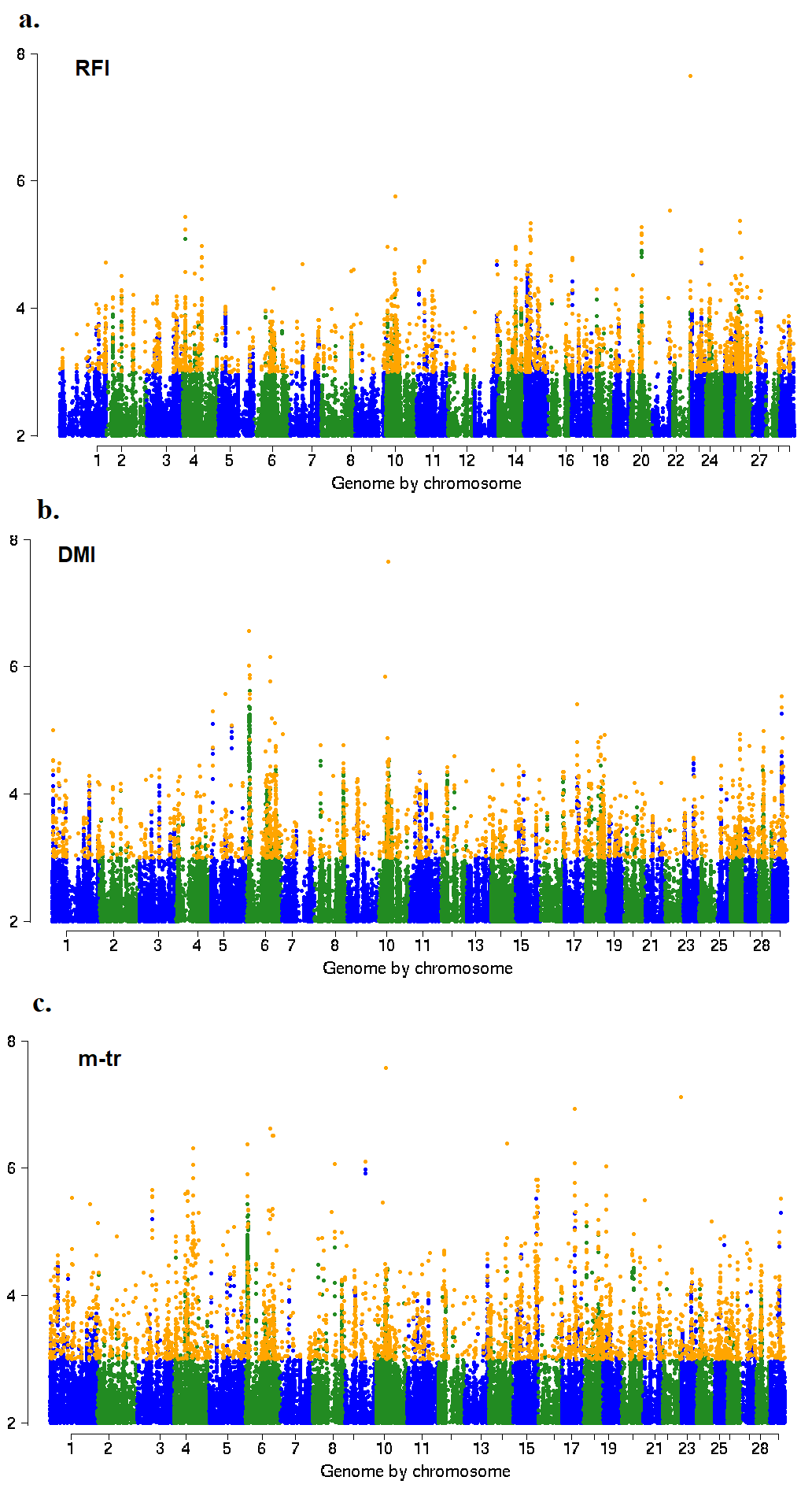

Supplement: Supplementary file 3 — Additional file 3: Figure S2. –log10(P-values) of single SNP regressions from single-trait GWAS for residual feed intake (RFI, a) and dry matter intake (DMI, b), and multi-trait meta GWAS (mt-tr, c) using the overseas (OVE) cow dataset. The orange points represent selected significant variants at P < 10–3. [file 12711_2022_749_MOESM3_ESM.png]
